# Aberrant immunomodulatory signature in β-propeller protein-associated neurodegeneration patient iPSC-derived microglia

**DOI:** 10.1038/s41598-026-55648-w

**Published:** 2026-06-15

**Authors:** Gamze Özata, Rachel M. Wise, Aida Cardona-Alberich, Naiyareen F. Mayeen, Stephan A. Müller, Stefan F. Lichtenthaler, Luigi Zecca, Lena F. Burbulla

**Affiliations:** 1 Metabolic Biochemistry, Biomedical Center (BMC), Faculty of Medicine, LMU Munich, Munich, Germany; 2 Graduate School of Systemic Neurosciences (GSN), LMU Munich, Munich, Germany; 3 Neuroproteomics, School of Medicine and Health, TUM University Hospital, Technical University of Munich, Munich, Germany; 4 German Center for Neurodegenerative Diseases (DZNE), Munich, Germany; 5 Munich Cluster for Systems Neurology (SyNergy), Munich, Germany; 6 Institute of Biomedical Technologies, National Research Council of Italy, Segrate (Milan), Italy; 7 Department of Metabolic Biochemistry, Faculty of Medicine, LMU Munich & German Center for Neurodegenerative Diseases (DZNE) Munich, Feodor-Lynen-Strasse 17, D-81377 Munich, Germany

**Keywords:** Neurology, Neuroscience

## Abstract

Microglia are the brain’s resident immune cells, essential for homeostasis and implicated in common neurodegenerative diseases like Alzheimer’s and Parkinson’s disease (PD), where their early activation and sustained inflammatory mediator release contribute to neuronal loss. However, their role in rare disorders is unclear. β-propeller protein-associated neurodegeneration (BPAN), caused by *WDR45* mutations, shares key features with PD, including iron accumulation and dopaminergic neuron loss, but the impact of microglia and mutant *WDR45* in BPAN pathophysiology remains unexplored. To address this, we established the first induced pluripotent stem stell (iPSC)-derived microglia model from BPAN patients. Parallel targeted transcriptomic and secretomic profiling revealed a shift from a homeostatic microglial toward a stress-adapted and transcriptionally reprogrammed state characterized by selective remodeling of immune signaling pathways and dysregulation of autophagy and cellular stress responses. Complementary secretomic analysis identified reduced secretion of lysosomal enzymes alongside increased shedding of immune-associated surface proteins, indicating altered lysosomal trafficking and remodeling of microglial immune signaling. These findings identify a distinct microglial phenotype in BPAN and implicate microglial dysfunction as a potential contributor to disease mechanisms, highlighting new avenues for therapeutic strategies targeting neuroimmune pathways.

**Supplementary Information:**

The online version contains supplementary material available at 10.1038/s41598-026-55648-w.

## Introduction

β-Propeller protein-associated neurodegeneration (BPAN) is a subtype of neurodegeneration with brain iron accumulation (NBIA), a family of ultra-rare neurological conditions^1–3^. This disorder is caused by primarily *de novo* mutations of the X-linked *WD repeat domain 45* (*WDR45*) gene which encodes the protein WDR45 (also called WIPI4) implicated in autophagy^4–6^. Analogous to autism spectrum and Rett syndrome-like disorders, pediatric BPAN patients often display epilepsy, developmental delays, cognitive impairment, and behavioral and language deficits^7^. The adolescent/adult phase is characterized by the onset of parkinsonism, dystonia, and dementia, evocative of Parkinson’s disease (PD) and Alzheimer’s disease (AD). Moreover, cellular-level pathological phenotypes including diffuse tau-positive neurofibrillary tangles^8^, thinning of the corpus callosum^9,10^, abnormal iron accumulation, and severe atrophy of dopaminergic neurons in the substantia nigra pars compacta (SNpc), suggest that BPAN may also share molecular mechanisms with these more common neurodegenerative diseases^2,8^.

Microglia are the resident immune cells of the central nervous system (CNS) critical for maintaining brain health and homeostasis through the complex orchestration of immune surveillance, synaptic pruning, debris clearance, and rapid responses to changes in the neuronal environment. In neurodegenerative diseases, microglia undergo dynamic transformations from surveillant to activated or “reactive” states, marked by the production of pro-inflammatory cytokines, altered phagocytic function, and increased antigen presentation. This shift can be protective in early stages, but when sustained, may promote chronic neuroinflammation and neurodegeneration^11^. Indeed, accumulating evidence from several neurodegenerative conditions has implicated dysregulated microglial function as an early and central player in disease progression, rather than a secondary response to neuronal death^12,13^. However, the role of microglia in rare neurodegenerative diseases remains underexplored.

Intriguingly, the SNpc contains one of the highest densities of microglia in the brain^14^, which react to aggregated forms of the PD-associated protein α-synuclein^15^ as well as neuromelanin-laden apoptotic neurons^16^ by releasing cytokines, chemokines, and other signaling molecules including reactive oxygen species (ROS), nitric oxide (NO), and complement factors^12^. In PD patient brains, excessive microglial activation precedes dopaminergic neuron death, and elevated microglial-derived inflammatory mediators have been observed in the SNpc, suggesting that reactive microglia may contribute to the preferential demise of these vulnerable neurons^17–25^. Most importantly, it has been shown that autophagy regulates microglial activation by inhibiting pro-inflammatory activity in resting conditions^26,27^, which makes the process of autophagy a key mediator of microglial-driven inflammation. Reports have shown that impaired microglial autophagy correlates with increased neuroinflammation, neuronal iron dyshomeostasis, and dopaminergic neuron death^26,28–31^. In vivo, knockout of autophagy genes specifically in microglia results in elevated neuroinflammation and worsened degeneration of nigral dopaminergic neurons, even manifesting the PD-like symptoms of impaired motor function and cognitive decline^32,33^. Interestingly, evidence of neuroinflammation in BPAN patients^9,10,34^, and elevated gliosis in one *Wdr45-*knockout mouse model^35^ indicate that aberrant microglial activity may be another shared pathology with PD, yet their potential involvement in BPAN pathology remains unexplored. This is particularly important as animal models of dopaminergic degeneration have shown that depleting microglia^36^ or inhibiting their pro-inflammatory activation^37–39^ results in attenuated destruction of this vulnerable neuron population, thus microglial modulation may represent a novel target for early therapeutic intervention and protection of dopaminergic neurons.

In vitro systems^40–43^ have provided valuable insight into the role of WDR45 in autophagosome initiation and elongation, and transgenic mouse models^35,44,45^ have elucidated the pathological consequences of defective *Wdr45* on both autophagic machinery and function in mouse neurons. However, existing cellular models do not recapitulate the unique complexity of dopaminergic neuron physiology, and available animal models do not embody important species-specific differences between mouse and human nigral dopaminergic neurons^46^. Thus, without proper manifestation of disease-relevant phenotypes in human brain cells, the precise roles of mutant *WDR45* in BPAN pathogenesis remain elusive. Advancements in induced pluripotent stem cell (iPSC) technology have facilitated the investigation of human neurons, and more recently glial cells^47^, providing an inimitable platform to define pathogenic phenotypes, probe for underlying mechanisms, and screen rescue strategies in patient cells. Indeed, the limited number of iPSC studies in BPAN have yielded valuable, complementary insights into disease mechanisms. One report using BPAN patient-derived dopaminergic neurons from iPSCs successfully recapitulated the iron accumulation observed in patient brains, alongside excessive oxidative stress and lysosomal dysfunction, suggesting that certain phenotypes may be intrinsic to WDR45-deficient neurons^48^. Similarly, another study established an iPSC-based midbrain dopaminergic neuron model from BPAN patients, which revealed defective autophagy, dysregulated gene expression in neurodegenerative and neurodevelopmental pathways, and responsiveness to cardiac glycosides that corrected autophagosome formation^49^. However, whether neuropathology is impacted by non-cell autonomous factors, and whether this impact is neuroprotective or neurotoxic in BPAN, has not yet been investigated.

In this study, we employed iPSCs derived from BPAN patients and healthy control subjects to investigate the cell autonomous consequences of loss of WDR45 function in microglia. Intriguingly, state-of-the-art approaches including high-performance secretome enrichment with click sugars (hiSPECS)^50^ and NanoString transcriptomic profiling revealed that BPAN patient-derived microglia undergo a shift from homeostasis toward dysregulation of immune, stress, and autophagy pathways, reduced secretion of lysosomal enzymes, consistent with potential lysosomal dysfunction, and enhanced shedding of antigen-presentation-related ectodomains - suggesting possible alterations in immune signaling. Together, these data suggest that microglial dysfunction may be involved in BPAN pathogenesis by sustaining a maladaptive neuroinflammatory environment, and should be further investigated as a potential component of the disease mechanism.

Our study presents the first patient-derived microglia platform for BPAN research and leverages this valuable tool to shed light on potential mechanisms that may contribute to BPAN neuropathology. We investigated how *WDR45* mutations alter microglial physiology and how these changes may contribute to non-cell autonomous vulnerability of dopaminergic neurons in the SN, taking critical steps towards a more holistic view of neuropathology in BPAN. Our findings lay the groundwork for advancing immunomodulatory research in BPAN and may open new avenues for therapeutic development targeting microglial dysfunction.

## Results

### iPSC-derived microglia from BPAN patients and healthy controls exhibit lineage identity and functional competence

To establish a disease-relevant, patient-derived model for studying microglial function in BPAN, we utilized iPSC lines from two individuals carrying heterozygous *WDR45* mutations, c.519+1_519 + 3delGTG (BPAN 1)^48^ and c.344 + 2T > G variant affecting the donor site of intron 5 (BPAN 2), alongside iPSC lines from two healthy controls (control 1, control 2) (Supplementary Dataset File 1). Of note, BPAN 1 patient carries an additional heterozygous *PLA2G6* variant (c.91G > A), however, its recessive inheritance pattern makes it unlikely to be pathogenic with a functional wild-type allele present. Thus, WDR45 deficiency is considered the primary cause of disease in this patient.

All four iPSC lines used in this study were thoroughly characterized for pluripotency prior to differentiation into microglia-like cells. Immunocytochemistry demonstrated robust expression of the canonical pluripotency markers SSEA and OCT4 (Supplementary Fig. 1A), as well as SOX2 and NANOG (Supplementary Fig. 1B). Quantitative PCR analysis further demonstrated high and comparable expression of key pluripotency genes, including *SOX2* (Supplementary Fig. 1C), *NANOG* (Supplementary Fig. 1D), and *TGDF* (Supplementary Fig. 1E) across all lines, collectively confirming that all iPSC lines maintained a pluripotent state.

iPSCs from BPAN patients and healthy controls were differentiated into microglia-like cells (iMG)^51,52^(Fig. 1A), exhibiting robust expression of canonical microglial markers, as confirmed by RT-qPCR analysis for *TMEM119*, and *P2RY12* (Supplementary Fig. 2A-B) or immunoblot analysis for IBA1 (Fig. 1B). These classical microglial markers were not detected in iPSC-derived dopaminergic neurons, which instead expressed the neuronal marker tyrosine hydroxylase (TH), absent in iMG of all four lines (Supplementary Fig. 2C).

**Fig. 1:**
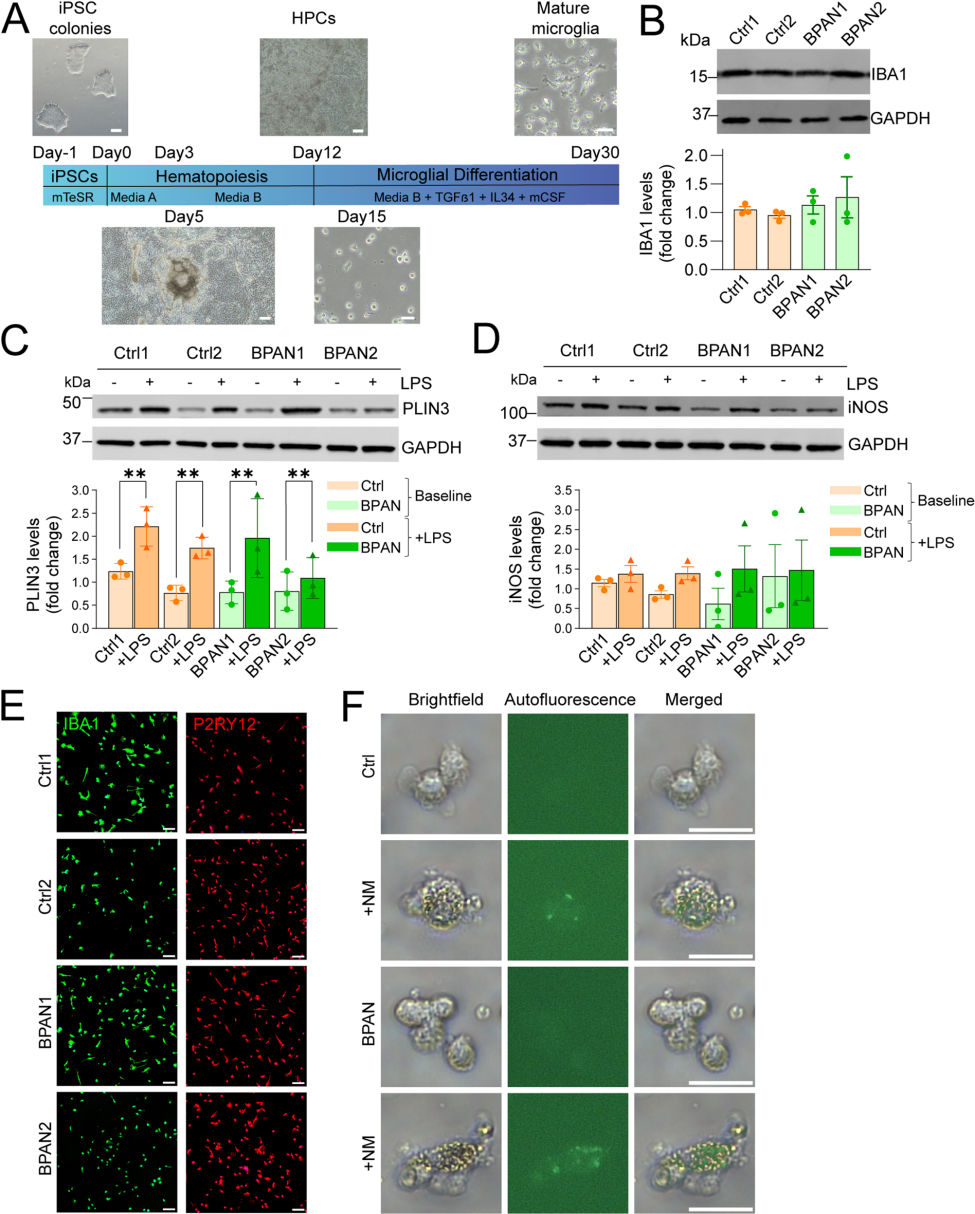
Differentiation and characterization of iPSC-derived microglia. (**A**) Schematic outline of the microglial differentiation protocol, with representative phase-contrast images of cultures at day 0, day 5, day 12, day 15, and day 30, illustrating key morphological transitions during maturation. (**B**) Western blot analysis of IBA1 protein expression across all iMG lines (*n* = 3 independent differentiations), with GAPDH as a loading control. Quantification is shown as bar plots (mean ± SEM). (**C**) Western blot analysis of PLIN3 protein levels in all lines, demonstrating a significant increase following LPS treatment (100 ng/ml, 24 h) compared to untreated controls with no significant differences observed between BPAN patient and control lines under baseline conditions. Statistical significance was determined by two-way ANOVA (*n* = 3 independent differentiations). (**D**) Western blot analysis of iNOS protein levels in all lines after LPS treatment (100 ng/ml, 24 h). Statistical significance was determined by two-way ANOVA showing no significant change compared to untreated controls or at baseline between cell lines (*n* = 3 independent differentiations). (**E**) Representative immunofluorescence images of all lines stained for IBA1 (green) and P2RY12 (red). Scale bar, 50 μm. (**F**) Representative images of control and BPAN patient iMG lines following neuromelanin treatment (5 ug/mL, 24 h), showing brightfield, neuromelanin autofluorescence, and merged channels. Scale bar, 10 μm. HPCs, Hematopoietic progenitor cells; NM, neuromelanin; LPS, lipopolysaccharide.

To assess the functional responsiveness of BPAN patient-derived iMG, we evaluated their ability to mount an innate immune response following inflammatory stimulation. PLIN3, a lipid-droplet surface protein marking lipid droplet-accumulating, pro-inflammatory microglia, and iNOS, an enzyme producing nitric oxide during innate immune activation, were selected as complementary indicators of inflammatory responsiveness^53,54^. Immunoblot analyses demonstrated that treatment with lipopolysaccharide (LPS), a bacterial endotoxin that activates innate immune responses, led to significant increase in PLIN3 protein levels (Fig. 1C) and persistent expression of iNOS (Fig. 1D), suggesting preserved inflammatory responsiveness. In addition, immunocytochemical analysis confirmed that iMG from both control and BPAN patient lines robustly expressed IBA1, a canonical marker of microglial identity (Fig. 1E, left panels in green). In parallel, P2RY12, a marker associated with homeostatic microglia, was also strongly expressed in all lines at baseline (Fig. 1E, right panels in red). The co-expression of IBA1 and P2RY12 validates the successful differentiation of iPSCs into iMG with a homeostatic phenotype, and while all lines robustly expressed these markers, minor morphological variability was observed, with BPAN 2 patient iMG appearing slightly more rounded and less ramified compared to the other lines.

To further interrogate microglial functionality in disease-relevant conditions, phagocytic competence was assessed by exposing iMG to neuromelanin isolated from human brain^55^. Neuromelanin is a physiological compound made of melanic, protein, lipid and metal components that accumulates in nigral dopaminergic neurons during healthy aging^56^. In PD, upon neuron death, neuromelanin is released into the extracellular space, then triggering activation of microglia which in the end degrade neuromelanin^57^. Brightfield and fluorescence microscopy demonstrated clear engulfment of exogenously provided neuromelanin, confirming preservation of phagocytic function and thus successful generation of mature, functional iMG from both control and BPAN patient iPSCs (Fig. 1F). In parallel, activation of the NLRP3 inflammasome, a multiprotein complex that senses cellular danger signals and triggers inflammatory responses, was evaluated by immunostaining. Under basal conditions, NLRP3 signal was low, however, following neuromelanin exposure, an increase in NLRP3 signal and the appearance of punctate structures were observed, a classic sign of inflammasome assembly (Supplementary Fig. 2D).

Together, these results demonstrate that differentiated iMG from both BPAN patients and controls exhibit key transcriptional, morphological, and functional hallmarks of mature microglia, providing a reliable and disease-relevant model system for downstream mechanistic investigations in BPAN.

## Transcriptomic profiling identifies a disease-associated immune signature in BPAN patient microglia

Transcriptomic profiling offers powerful insights into disease-associated molecular changes. In BPAN, the transcriptomic changes underlying neurodegeneration remain poorly understood. To address this gap, we performed transcriptomic analysis on iMG from BPAN patients and healthy control lines using the NanoString platform (Supplementary Dataset File 2), which enables the sensitive detection of immune, apoptotic, and neuroinflammation-related gene expression changes.

Targeted transcriptomic profiling using the Neuroinflammation gene panel revealed significant alterations in gene expression related to key microglial pathways in BPAN patient iMG compared to controls (Supplementary Dataset File 2). Heatmap visualization highlighted widespread changes across key microglial pathways including innate and adaptive immunity, cytokine signaling, stress response, microglial homeostasis, and apoptosis, indicating a loss of homeostatic signature (Fig. 2A).

**Fig. 1:**
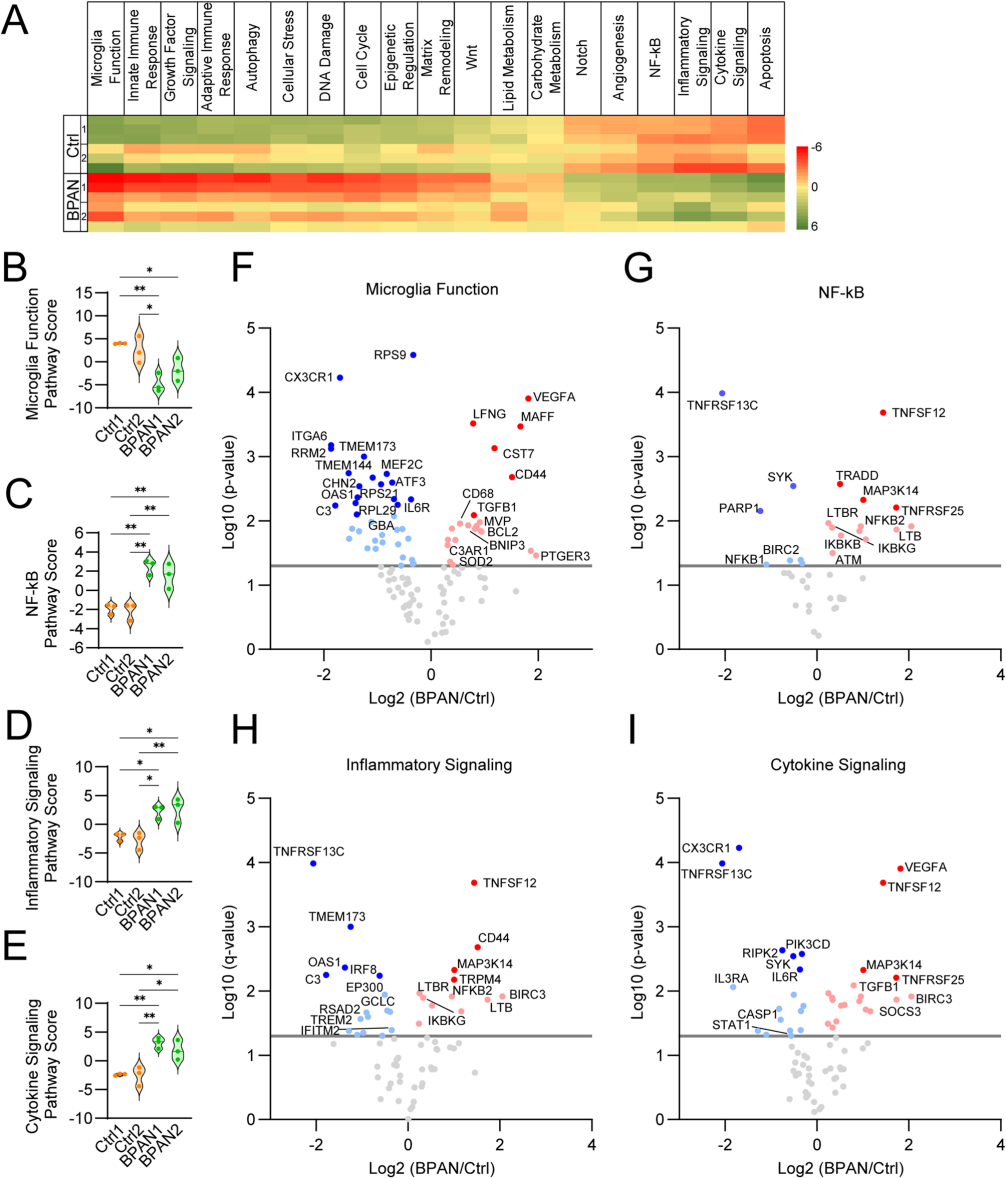
NanoString-based neuroinflammatory pathway analysis shows proinflammatory phenotype. (**A**) Heatmap displaying pathway scores for 19 relevant neuroinflammation-associated pathways, derived from the NanoString Neuroinflammation Panel, across individual samples (three replicates per line; two control and two BPAN patient lines). Red indicates pathway downregulation and green indicates upregulation relative to baseline (*n* = 3 independent differentiations with 2 iPSC lines per control/patient group). (**B–E**) Violin plots showing pathway scores for selected functional categories, including microglia function (**B**), NF-κB signaling (**C**), inflammatory signaling (**D**) and cytokine signaling (**E**). Each violin plot displays the distribution of pathway scores for all samples in each group (*n* = 3 independent differentiations). Statistical significance was determined by one-way ANOVA. (**F-I**) Volcano plots illustrating differential gene expression within the most relevant pathways: microglia function (**F**), NF-κB signaling (**G**), inflammatory signaling (**H**), and cytokine signaling (**I**). Genes passing Benjamini–Hochberg FDR correction are displayed as dark red dots (upregulated) and dark blue dots (downregulated). Genes with nominal significance (*p* < 0.05), but not passing FDR correction, are shown in light red (upregulated) and light blue (downregulated) dots. Light gray dots represent nonsignificant changes. The gray horizontal line marks the significance threshold with p-values (*p* = 0.05, –log₁₀ = 1.3).

Given the limited number of donor lines (two BPAN patient and two control lines), genotype is inherently confounded with cell line. Therefore, statistical estimates are interpreted as reflecting differences between BPAN patient and control donor groups at the level of the included lines, rather than independent population-level genotype effects.

Despite this limitation, multiple genes exhibited concordant directional changes across both BPAN patient lines relative to both controls, supporting a consistent disease-associated transcriptional signature.

Significant differences in BPAN patient iMG were identified through pathway score analysis using the NanoString Advanced Analysis module, which summarizes the activity of predefined biological pathways based on coordinated expression patterns across grouped target genes, rather than relying solely on individual gene-level changes. Core microglial programs were disrupted, with downregulation of genes associated with microglial homeostasis and surveillance (Fig. 2B), and upregulation of pathways involved in NF-kB signaling, cytokine production, and inflammatory activation (Fig. 2C–E). For descriptive pathway-level analyses, genes with nominal differential expression (*p* < 0.05) were examined to evaluate coordinated patterns within signaling pathways, while FDR-adjusted q-values were used to determine statistical significance. Notably, the dysregulation of autophagy-related genes (Supplementary Fig. 3A, E) is consistent with the known role of WDR45 in autophagic regulation.

To further dissect these pathway-level changes, we focused on specific genes within these dysregulated networks. Core microglial maintenance genes (*CX3CR1*, *MEF2C*) were downregulated, while genes linked to inflammation and stress responses, such as *CST7*,* VEGFA*,* CD44 and PTGER3*, were upregulated (Fig. 2F). Collectively, these changes indicate a transcriptionally reprogrammed microglial state characterized by loss of homeostatic identity and acquisition of stress-associated features.

Further, genes involved in microglial homeostasis, immune resolution, and phagocytosis (*ITGA6*,* OAS1*,* GBA*,* C3AR1*) were altered, alongside widespread downregulation of ribosomal genes, indicating impaired protein synthesis and a metabolically compromised state (Fig. 2F). Simultaneously, stress adaptation and survival genes (*SOD2*,* MVP*,* TGFB1*,* BCL2*,* BNIP3*) were elevated, consistent with chronic cellular stress and enhanced survival signaling.

The NF-κB pathway demonstrated selective engagement, characterized by upregulation of TNF superfamily ligands and receptors (*TNFSF12*,* LTB*,* LTBR*,* TNFRSF25*) and intracellular mediators (*MAP3K14*,* IKBKB*), whereas *NFKB1* (p50) was reduced, suggesting remodeling of canonical NF-κB signaling dynamics rather than uniform pathway activation. Notably, increased expression of *MAP3K14* (*NIK*) and *NFKB2* is consistent with preferential activation of the non-canonical NF-κB pathway, which is typically associated with sustained TNF-family receptor signaling and transcriptional reprogramming (Fig. 2G).

Microglial signaling was further altered by suppression of key regulators of immune homeostasis (*TREM2*,* C3*,* OAS1*,* IL3RA*,* IRF8*) and altered cytokine receptor expression, including decreased *IL6R* and *IL3RA*, indicating modified responsiveness to interleukin-mediated signaling (Fig. 2H, I). In parallel, multiple interferon-responsive genes (*OAS1*,* RSAD2*,* STAT1*,* IFITM2*) were reduced, suggesting attenuation of antiviral and interferon-driven programs (Fig. 2H). Also, concurrent upregulation of genes such as *CD44*, *VEGFA*, and *SOCS3* further supports a state of immune remodeling characterized by stress adaptation and regulatory feedback (Fig. 2H, I).

Autophagy-related genes displayed a mixed regulatory pattern, with compensatory upregulation of *ATG7* contrasted by downregulation of key mediators involved in autophagosome maturation and mitophagy (*RB1CC1*,* CLIC4*,* PINK1*) indicating potential impairment in autophagosome maturation and lysosomal degradation. Similarly, reduced expression of *CLEC7A*,* SYK* and *PROS1* points to disrupted crosstalk between innate immune signaling and autophagy, which may compromise microglial phagocytic capacity and hinder effective clearance of cellular debris. (Supplementary Fig. 3E).

A transcriptional shift consistent with apoptosis resistance and chronicity rather than acute inflammatory cell death was indicated by the downregulation of pro-apoptotic mediators (*CASP3*,* CASP1*) alongside upregulation of anti-apoptotic genes (*BCL2*,* BIRC3*) (Supplementary Fig. 3B, F). This pattern was further supported by increased expression of survival-associated regulators such as *MDM2* and *BNIP3*, and reduced levels of pro-apoptotic mediators including *BIRC2* (Supplementary Fig. 3F), which was accompanied by reduced *GCLC* (Fig. 2H) suggesting diminished oxidative stress resilience. DNA damage response pathways showed broad dysregulation (Supplementary Fig. 3C), with downregulation of homologous recombination genes such as *RAD51*,* RAD51C*, and *BARD1*, along with checkpoint mediators *RAD17* and *H2AFX*, suggesting impaired double-strand break repair (Supplementary Fig. 3G). Although upstream sensors *ATR* and *ATM* were upregulated, downstream repair signaling appeared insufficiently activated, indicating compromised genome maintenance capacity, potentially favoring genomic instability and persistent cellular stress. Consistent with DNA damage, the cell cycle pathway showed an overall decrease (Supplementary Fig. 3D), with key regulators of DNA replication and mitotic progression such as *RRM2*, *KIF2C*, and *PCNA* downregulated (Supplementary Fig. 3H), suggesting impaired proliferative capacity and cell cycle progression.

Given that the BPAN patient iPSC lines were derived from female donors while controls were from male donors, we next evaluated potential sex-specific effects on the observed microglial transcriptomic profiles. We performed a dedicated sex-aware sensitivity analysis using published sex-differential microglial gene signatures^58^(Supplementary Dataset File 3). Gene-level directionality comparisons showed no systematic concordance between sex-associated and BPAN patient-associated transcriptional changes, and enrichment analyses demonstrated that sex-differential genes were not overrepresented among NanoString differential-expression hits.

These findings suggest that the observed transcriptional differences are not strongly explained by known sex-biased gene expression signatures. However, given that sex and genotype are fully confounded in the current study design, a contribution of donor sex cannot be excluded, and the observed disease-associated signature should be interpreted within this context.

In summary, BPAN patient iMG exhibit a deeply altered transcriptomic signature that reflects a shift from homeostatic microglial functions toward a stress-adapted and reprogrammed state. This signature is characterized by loss of key homeostatic markers and selective remodeling of immune signaling pathways. Together, these changes suggest a chronically recalibrated microglial baseline that may alter neuron-microglia communication and functional responses in BPAN.

### Secretome analysis reveals reduced secretion of lysosomal enzymes, altered shedding of antigen-presentation–related ectodomains, and chronic activation of BPAN patient microglia

**Fig. 3:**
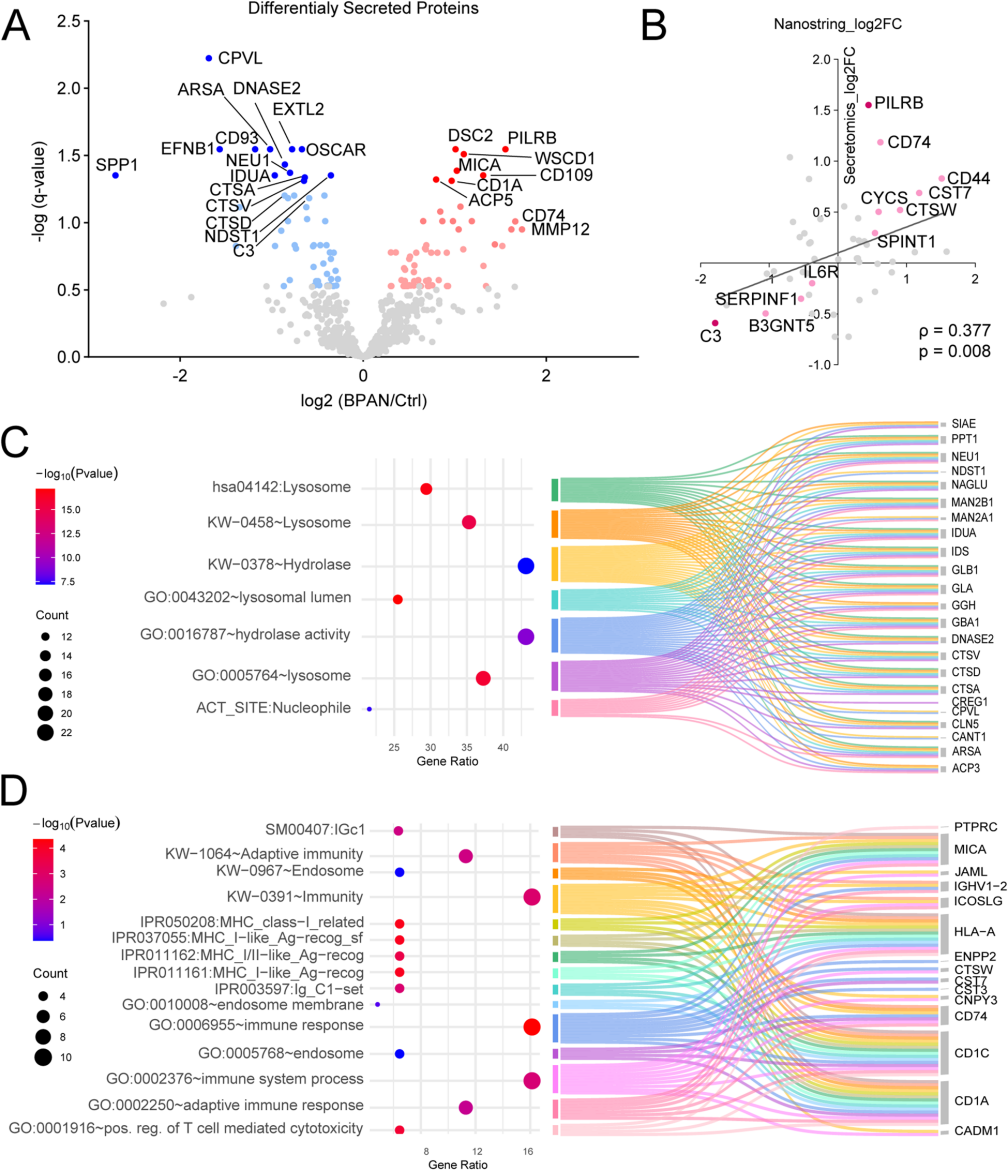
Secretomics analysis of BPAN patient iPSC-derived microglia. (**A**) Volcano plot of BPAN patient versus control iMG. The -log10 transformed q-values of each protein are plotted against their log2 fold change transformed protein label free quantification ratios. Proteins significantly more secreted from BPAN patient iMG after Benjamini–Hochberg FDR correction are displayed as dark red filled dots, while less secreted proteins are displayed as dark blue filled dots. Proteins with nominal significance (*p* < 0.05), but not passing FDR correction, are shown in light red (more secreted) and light blue (less secreted). Unaltered proteins are displayed as gray dots. Proteins are labeled with their UniProt gene names. (**B**) Scatter plot comparing log₂ fold-change (log₂FC) from the NanoString RNA panel (x-axis) and secretome proteomics (y-axis) for targets quantified in both datasets. Tier 1 genes (dark pink) were significant in both RNA and protein layers after Benjamini–Hochberg FDR correction (q < 0.10), Tier 2 genes (light pink) were significant based on a directional Stouffer–integrated RNA–protein statistic with concordant fold-change direction (q < 0.05), and Tier 3 genes (gray) did not meet these criteria. The line indicates the linear trend, and overall RNA–protein concordance was assessed by Spearman correlation (ρ = 0.377, *p* = 0.008). (**C**) Group of terms in cluster 1 of Pathway enrichment analysis for downregulated proteins (*p* < 0.05). The dot plots (left) show the total gene number of a term in percentage as dot size and the p-values as a color gradient. Sankey plots (right) show how each term relates to each gene. (**D**) Group of terms in cluster 4 of Pathway enrichment analysis for upregulated proteins (*p* < 0.05). The dot plots (left) show the total gene number of a term in percentage as dot size and the p-values as a color gradient. Sankey plots (right) show how each term relates to each gene.

To assess the paracrine signaling of BPAN patient iMG, we employed hiSPECS, a sensitive mass spectrometry-based approach that enables selective profiling of secreted glycoproteins^50^. This method provides a comprehensive and unbiased snapshot of the secretory activity of living cells under physiological conditions, capturing even low abundance secreted factors (Supplementary Dataset File 4). Secretome profiling of BPAN patient iMG revealed several converging alterations with potential functional relevance (Fig. 3A).

Statistical analyses were performed on LC–MS/MS measurements; however, given the nested structure of the data, these results are interpreted in the context of donor-line-level comparisons. Accordingly, significance reflects differences between BPAN patient and control groups as represented by the included donor lines.

Notably, many of these proteins showed consistent directional changes across both BPAN patient lines, further supporting the robustness of the observed phenotype.

Most prominently, proteins involved in lysosomal degradation and phagocytic processes were broadly less abundant in the secretome of BPAN patient iMG. This included acid hydrolases such as ARSA, IDUA and the cathepsins CTSA and CTSV. This pattern could indicate a reduced capacity for degrading phagocytosed material and clearing cellular debris, or altered lysosomal trafficking and secretion dynamics suggesting compromised major microglial housekeeping functions.

In parallel, there was a substantial reduction of the marker osteopontin (SPP1) in patient iMG, which is typically elevated in other neurodegenerative conditions and has been linked to neuroprotective and reparative functions^59^. Reduced SPP1 secretion may reflect impaired engagement of reparative or neuroprotective signaling programs.

Conversely, several immune-associated surface proteins showed a higher abundance in BPAN patient iMG secretome. Significant increases were observed for CD1A and MICA, both of which can be released via proteolytic ectodomain shedding. In addition, CD74 and MMP12 showed nominally significant increases, further supporting altered immune-associated surface remodeling and extracellular matrix regulation. Together, these findings indicate increased shedding of immune-associated surface molecules and remodeling of antigen presentation-related components, consistent with altered microglial immune interface signaling.

Integration of transcriptomic (Fig. 2) and secretomic data (Supplementary Fig. 4) identified 49 overlapping genes with defined log2 fold-change values in both datasets (Supplementary Dataset File 5). Across these genes, RNA and protein log2 fold changes showed a significant positive association (Spearman ρ = 0.377, *p* = 0.008), indicating coordinated transcriptional and secreted protein regulation between control and BPAN patient iMG (Fig. 3B). Of note, the secretomic data here includes both directly secreted proteins and proteins released by shedding adding another level of complexity. A permutation analysis (10,000 random gene-label shuffles) demonstrated that the observed correlation exceeded the 95th percentile of correlations expected under random pairing (ρ₉₅% = 0.235; empirical *p* = 0.0032), supporting non-random RNA-protein concordance. Directional analysis showed that 35 of 49 genes (71.4%) changed in the same direction at both RNA and protein levels, whereas 14 genes (28.6%) were discordant. To distinguish confidence levels, overlapping genes were stratified into evidence tiers based on statistical significance in each platform.

Two genes, C3 and PILRB, met stringent confirmatory criteria (Tier 1), demonstrating significant combined evidence and concordant directional changes across platforms. C3 was reduced at both levels, whereas PILRB showed coordinated upregulation, suggesting cross-platform regulation of complement-related and immune receptor pathways in BPAN patient iMG. In addition, nine genes met exploratory-level support (Tier 2), i.e. CST7, B3GNT5, CD44, CD74, CTSW, CYCS, SERPINF1, IL6R, and SPINT1, all of which exhibited concordant directional changes between RNA and protein (Supplementary Fig. 4). While not all reached statistical significance on both platforms individually, the consistent directionality and significant combined statistics support coordinated transcriptional and secretory regulation of these pathways.

Together, these findings indicate that a defined subset of immune-associated and stress-related genes exhibit non-random RNA-protein coupling in BPAN patient microglia, reinforcing the presence of structured transcriptional-secretory reprogramming.

To determine whether RNA-protein integration was driven by a specific glycoprotein class enriched by hiSPECS, we stratified secretome proteins based on curated UniProt annotations (Supplementary Dataset File 4). Using a hierarchical approach, proteins annotated as lysosomal lumen were first assigned to a lysosomal group, followed by secreted proteins (signal peptide, no transmembrane domain), and finally remaining single-pass/type-I transmembrane proteins assigned to a transmembrane (TM) group, generating mutually exclusive categories. RNA-protein correlations were observed across lysosomal and secreted proteins and were weaker for transmembrane proteins; however, statistical comparisons revealed no significant differences in correlation strength between classes. These findings indicate that the observed RNA-protein coupling is not attributable to a single enriched biological category but is broadly distributed across secreted and lysosomal proteins.

Follow-up pathway enrichment analysis of secreted proteins from BPAN patient and control iMG revealed several alterations to functionally distinct clusters (Fig. 3C, D, Supplementary Dataset File 6). Cluster 1, comprising proteins reduced in BPAN patient iMG secretome, was enriched for lysosomal components and enzymes essential for macromolecule degradation like hydrolases (Fig. 3C). Key lysosomal hydrolases such as CTSD, GLB1, IDUA, IDS, GBA1, NAGLU and DNASE2 were among the proteins showing reduced secretion. Additional lysosomal enzymes including MAN2A1, MAN2B1, NEU1, NDST1, CTSA and CTSV further shows the reduced abundance of hydrolases within this cluster. These alterations potentially indicate impaired lysosomal function, consistent with compromised autophagic clearance and defective processing of phagocytosed material, key processes for microglial maintenance of neural tissue homeostasis. Proteins with higher abundance in BPAN patient iMG secretome (Cluster 4) were strongly enriched for immune-related pathways, including adaptive immune response, antigen recognition domains, positive regulation of T cell mediated cytotoxicity, and broader immune response processes (Fig. 3D). For example, CD74, CD1A and CD1C, key molecules involved in antigen shedding, reflect possible alterations in immune signaling. Correspondingly, the enrichment of MHC class I-related and MHC-like antigen recognition domains, including HLA-A, potentially indicate altered antigen-presentation-related signaling. The higher abundance of immune surveillance ligand MICA, protease inhibitors CST3, CTSW and CST7, immune modulator JAML, along with receptor PTPRC (CD45), collectively reflects enhanced regulation of extracellular proteolysis, immune signaling, and cell-cell interactions in this cluster.

Together, these findings define a distinct secretory profile in BPAN patient iMG, marked by reduced secretion of lysosomal enzymes, consistent with potentially impaired lysosomal and phagocytic function, and a differentially activated state with increased shedding of antigen-presentation-related ectodomains that may contribute to neuroinflammatory pathology.

## Discussion

Our multi-layer analysis of BPAN patient-derived iMG reveals a complex and divergent activation profile expanding BPAN research beyond neuronal and autophagic pathways to highlight the importance of glial contributions and neuroimmune mechanisms. Notably, this profile includes transcriptional downregulation of homeostatic genes such as *CX3CR1* and *MEF2C*, crucial for synaptic pruning and neuron-microglia communication^60,61^. In parallel, upregulation of inflammatory and remodeling genes like *CD44* and *VEGFA* together with downregulation of the complement component *C3* indicates restructuring of immune and extracellular signaling pathways rather than a clear shift toward a reactive, neuroinflammatory phenotype^62,63^. In addition, downregulation of *IRF8* disrupts homeostatic maintenance, phagocytic function, and inflammatory regulation^64,65^. These changes suggest a microglial state that may impair neuronal function or exacerbate neurodegeneration.

Further, analysis of NF-κB signaling showed evidence of selective pathway engagement in BPAN patient iMG versus healthy controls. NF-κB signaling is highly context-dependent within the CNS, with distinct functional outcomes across cell types. While microglial NF-κB activity is commonly linked to pro-inflammatory responses, neuronal NF-κB has been associated with neuroprotective functions, including regulation of cell survival and synaptic plasticity^66–68^. Upregulation of *MAP3K14* and *IKBKB*, along with increased expression of TNF-superfamily ligands such as *TNFSF12* (*TWEAK)*, *LTB* and its receptor *LTBR*, supports remodeling of NF-κB–associated pathways that are known to influence microglial activation in inflammatory and neurodegenerative contexts^69,70^. Mechanistically, these differences are partly mediated by the balance between canonical (*NFKB1/RELA*-driven) and non-canonical (*NFKB2/MAP3K14*-dependent) pathways, which regulate acute versus sustained transcriptional programs, respectively^71,72^. Consistent with this framework, the observed decrease in *NFKB1* alongside increased *MAP3K14* and *NFKB2* suggests a shift toward non-canonical NF-κB signaling, potentially promoting a chronically reprogrammed microglial state rather than a transient inflammatory response. Together, these findings indicate reshaping of NF-κB signaling dynamics.

Moreover, downregulation of antiviral genes like *OAS1* and *RSAD2* suggests impaired innate immune defense, likely reducing their ability to detect and respond to viral pathogens with type I interferon signaling^73^. Upregulation of survival genes such as *BCL2* and *BIRC3* together with activation of NF-κB signaling and increased expression of factors such as TGFB1 and VEGFA, indicates a shift toward an apoptosis-resistant, senescence-like state^74^. This transcriptional pattern may lead to the persistence of dysfunctional microglia in the brain, contributing to chronic neuroinflammation.

Lastly, altered expression of autophagy-related genes (*ATG7*,* CLEC7A*,* PROS1*) may reflect compensation for WDR45-associated autophagy dysfunction, supporting the idea that defective clearance mechanisms may contribute to the progression of neurodegeneration in BPAN^35,43^.

To further explore the functional consequences of the transcriptional changes in BPAN patient iMG, we assessed whether dysregulated gene expression translated into altered protein secretion, which is critical for understanding microglial communication and effector function. In fact, BPAN patient iMG displayed a distinct secretory signature marked by altered lysosomal enzyme release, immune interface remodeling, and stress-associated changes.

The coordinated low abundance secretion of lysosomal enzymes, including CTSD, GLB1, IDUA, and NEU1, alongside the suppression of the complement component C3, indicates altered lysosomal trafficking and complement regulation suggesting impaired microglial clearance of apoptotic material and protein aggregates – a hallmark of microglial dysfunction in neurodegenerative diseases^75,76^ and a process critical for maintaining CNS homeostasis and preventing inflammation^1,41,77^. These lysosomal deficits may reflect a disruption in autophagy-linked exocytosis, a cellular process previously shown to be impaired in WDR45-deficient non-microglial cells^35,41,48^ and here demonstrated for the first time in patient-derived microglia.

The increased secretion of immune mediators, including antigen-presentation-related ectodomains CD74, MICA, CD1A, as well as protease MMP12, suggests a shift toward enhanced shedding of immune-associated surface molecules, which could influence or prolong local neuroimmune signaling in BPAN. These changes may represent either a compensatory attempt to respond to cellular damage or a maladaptive immune remodeling driven by lysosomal stress. When considered alongside the altered lysosomal enzyme secretion, this dual signature of dysfunction, combining defective degradative capacity with remodeled immune alertness, supports a direct contribution of microglia to BPAN pathology. These findings further support emerging models positioning dysfunctional microglia as central players in neurodegeneration, reinforcing the rationale for therapeutic strategies aimed at restoring microglial homeostasis and function. The coordinated reduction of complement component C3 together with increased inhibitory receptor PILRB expression suggests a shift in the balance of microglial immune sensing, possibly favoring restrained activation over complement-mediated engagement. In parallel, concordant regulation of stress- and lysosome-associated genes such as CST7 and immune surface molecules including CD74 supports restructuring of immune interface signaling at both transcriptional and secretory levels. These changes point toward chronic immune recalibration likely driven by autophagy impairment. Functionally, this state may compromise efficient debris clearance and alter neuron-microglia communication, thereby contributing to progressive vulnerability in BPAN.

While the present study provides the first-of-its-kind transcriptomic and secretomic characterization of BPAN patient-derived iMG, several limitations should be acknowledged. First, BPAN patient and control iPSC lines in this study were not sex-matched. Because sex differences in microglial gene expression have been reported^58,78^, this mismatch represents a potential source of confounding that must be considered when interpreting differential-expression results. While our sensitivity analysis did not indicate strong overlap between sex-associated and BPAN-associated transcriptional changes, the complete confounding of sex and genotype in the current study design prevents definitive separation of these effects. Future studies using sex-matched donor lines will be required to fully disentangle genotype- and sex-specific contributions. In addition, findings in this study are derived from a limited number of iPSC lines, i.e. two BPAN patient and two healthy control donor lines. Future studies employing larger and fully sex-matched cohorts will be important to further validate disease-specific effects and to fully disentangle WDR45-driven biology from sex-associated transcriptional variation.

Second, iPSC-derived microglia are powerful tools, but may not fully capture the complexity of microglial development and function within the 3D CNS environment, including interactions with neurons, astrocytes, vasculature, and the extracellular milieu. Third, the cross-sectional nature of our data does not address dynamic changes over time or the bidirectional nature of microglial communication that shape their phenotype and function in vivo. Also, while transcriptomic and secretomic profiles provide insight into altered pathways, further functional validation including LysoTracker quantification, autophagic flux assays, and flow cytometry for surface markers will be required to establish causal links between specific dysregulated programs and disease progression. Finally, we note that transcriptomic profiling used a targeted neuroinflammation panel with a pre-selected genes rather than whole-transcriptome sequencing, and secretomic profiling captured extracellular glycoproteins via hiSPECS enrichment rather than total cellular proteome. Therefore, integration of these datasets should be interpreted as exploratory and used to identify candidate signals for future functional validation. Addressing these limitations in future studies, for example, by incorporating co-culture or organoid models, performing functional assays, and correlating findings with in vivo models, will be important to further define the contribution of microglia to BPAN pathogenesis.

To date, BPAN research has focused predominantly on neuronal and autophagy-related mechanisms, with relatively little attention paid to the role of glia or the neuroimmune system. By shifting focus to patient-derived microglia, our study begins to fill this gap, demonstrating that BPAN patient iMG display a dysregulated molecular signature. Overall, our study emphasizes that BPAN patient iMG exhibit features consistent with a chronic maladaptive inflammatory state and should be considered as likely contributors to neurodegeneration and promising therapeutic targets for future investigations.

## Materials and methods

### Subjects

Included in this study are iPSCs from two female BPAN patients (BPAN 1 = L-8172^48^; BPAN 2 = 119129, registered at https://biobanknetwork.telethon.it/Sample/View?sampleId=119129) and two healthy male control individuals (control 1 = SFC156-03-012, registered at hPSCreg: https://hpscreg.eu/cell-line/STBCi101-A; control 2 = SFC065-03-05, registered at hPSCreg: https://hpscreg.eu/cell-line/STBCi057-B). The study was approved by the Ethics Committee of the Medical Faculty of LMU Munich, Germany, and all participants gave written informed consent. All methods were performed in accordance with the relevant guidelines and regulations.

### Culture and characterization of human iPSCs

iPSC cultures were grown as colonies and maintained on Cultrex-coated (R&D Systems) plates and antibiotic-free mTESR Plus medium (Stem Cell Technologies) was refreshed every other day. Cells were manually passaged every 5–7 days.

BPAN patient and control iPSC lines were characterized for expression of pluripotency markers by quantitative RT-PCR (SOX2, NANOG, TDGF1). RNA was extracted from subconfluent iPSC colonies using the RNeasy kit (Qiagen), then reverse transcribed into cDNA using the iScript™ cDNA Synthesis Kit (Biorad). Quantitative RT-PCR was performed with SYBR GreenER (Thermo Scientific) on the 7500 Fast Real-Time PCR system (Applied Biosystems). Primer sequences were designed with Primerblast and purchased from Integrated DNA Technologies (Table 1). Ribosomal protein L13 (RPL13) was used as housekeeping gene. All lines were routinely tested for mycoplasma contamination.

**Table 1:** List of RT-qPCR primers used for pluripotency validation of iPSC lines.

Primer name	Sequence 5’-3’
h-SOX2-F	GGG AAA TGG GAG GGG TGC AAA AGA GG
h-SOX2-R	TTG CGT GAG TGT GGA TGG GAT TGG TG
h-TDGF-F	CAG ATC CTA AAC AGC TCG CAG AAT
h-TDGF-R	GCG TAC GCA AAT TAA AGT CCA GA
h-NANOG-F	CAG CCC CGA TTC TTC CAC CAG TCC C
h-NANOG-R	CGG AAG ATT CCC AGT CGG GTT CAC C
h-RPL13-F	CAT AGG AAG CTG GGA GCA AG
h-RPL13-R	GCC CTC CAA TCA GTC TTC TG

## Differentiation and characterization of human iPSC-derived microglia culture

iPSC-derived microglia (iMG) were generated from BPAN patients and controls according to the protocol by McQuade et al., 2018 with modifications^51,52^. Briefly, 4–6 medium-sized iPSC colonies were manually seeded onto a Cultrex-coated six-well plate with fresh mTeSR™ Plus medium. After 18–24 h, wells containing approximately 4–10 attached colonies/cm² were used for hematopoietic differentiation. Hematopoietic progenitor cells (HPCs) were generated using the STEMdiff hematopoietic kit (STEMCELL Technologies) following the manufacturer’s protocol for 12 days. On day 12, HPCs were gently harvested and seeded into 6-well plates at a density of 1–2 × 10^5 cells per well in iMG differentiation medium (phenol-free DMEM/F12 supplemented with 2X B27 (Thermo Fisher Scientific), 2X insulin-transferrin-selenite (Thermo Scientific), 0.5X N2 (Life Technologies), 1X GlutaMAX (Life Technologies), 1X MEM non-essential amino acids solution (Thermo Scientific), 5 µg/mL human insulin (Sigma Aldrich), and 400µM α-thioglycerol (Sigma Aldrich). The microglia differentiation medium was supplemented with the following factors for microglia lineage commitment: IL-34 (100 ng/ml; Peprotech), TGF-β1 (50 ng/ml; Peprotech), and M-CSF (25 ng/ml; Peprotech). iMG were maintained in this media until day 30, when the cells were considered mature.

BPAN patient and control iMG were characterized by quantitative RT-PCR (TMEM119, P2RY12). RNA extraction was performed using the RNeasy kit (Qiagen) which was then reverse transcribed into cDNA with the iScript ™ cDNA Synthesis Kit (Biorad). Quantitative RT-PCR was performed with SYBR GreenER (Thermo Scientific) on the 7500 Fast Real-Time PCR system (Applied Biosystems). Primer sequences were designed with Primerblast and purchased from Integrated DNA Technologies (Table 2). ALU repeat units was used as housekeeping gene.

**Table 2:** List of RT-qPCR primers used for validation and characterization of iPSC-derived microglia.

Primer name	Sequence 5’-3’
h-TMEM119-F	CGG CCT ATT ACC CAT CGT CC
h-TMEM119-R	CTG GGC TAA CAA GAG AGA CCC
h-P2RY12-F	CAC TGC TCT ACA CTG TCC TGT
h-P2RY12-R	AGT GGT CCT GTT CCC AGT TTG
h-TH-F	CGG GCT TCT CGG ACC AGG TGT A
h-TH-R	TCC TCG GCG GTG TAC TCC ACA
h-ALU-F	CAT GGT GAA ACC CCG TCT CTA
h-ALU-R	GCC TCA GCC TCC CGA GTA G

## Differentiation of human iPSCs into midbrain dopaminergic neurons

The differentiation of midbrain dopaminergic neurons from iPSCs was done as previously described with minor adaptations^79^. In detail, healthy control human iPSCs were cultured on Cultrex-coated plates in antibiotic-free mTeSR Plus medium (StemCell Technologies) and manually passaged every 5–7 days. Prior to passaging, spontaneously differentiated regions were removed under a stereomicroscope, and colonies were mechanically fragmented using a pipette tip. Aggregates were transferred to fresh Cultrex-coated wells and maintained at 37 °C with 5% CO₂, with medium changes every 48 h. Neuron differentiation was initiated when cultures reached over 95% confluence (Day 0). On day 0, the medium was changed to KSR differentiation medium composed of KnockOut DMEM (Thermo Fisher), 15% KnockOut Serum Replacement (Thermo Fisher), 1× GlutaMAX (Thermo Fisher), 1× penicillin-streptomycin (Thermo Fisher), and 1 mM β-mercaptoethanol (Sigma-Aldrich), supplemented with dual SMAD inhibitors LDN193189 (LDN) and SB431542 (SB). On day 1, Sonic hedgehog protein (SHH), purmorphamine, and Fibroblast Growth Factor 8 (FGF8) were added, followed by CHIR99021 (CHIR) on day 3 to promote floor plate patterning. On day 5, cells were transitioned to NbSm1 medium, consisting of Neurobasal Medium (Thermo Fisher), NeuroCult SM1 supplement (STEMCELL Technologies), 1× GlutaMAX (Thermo Fisher), and 1× penicillin-streptomycin (Thermo Fisher), supplemented with LDN193189, SHH, purmorphamine, FGF8, and CHIR. From day 7, SHH, purmorphamine, and FGF8 were withdrawn, and only LDN and CHIR were maintained. Between days 11–14, cells were passaged as 1–2 mm chunks onto poly-D-lysine/laminin-coated dishes in NbSm1 medium supplemented with CHIR, BDNF, ascorbic acid, GDNF, TGF-β3, dibutyryl-cAMP, and DAPT. Between days 25–30, cells were dissociated into single-cell suspensions using Accutase and replated on coated dishes in the same media formulation. From day 40 onward, cells were maintained in NbSm1 medium without supplements until collection.

### Immunofluorescent staining and image analysis

For immunocytochemical analysis, all iPSCs lines were seeded onto Cultrex-coated (R&D Systems) glass coverslips and iMG were seeded onto uncoated glass coverslips, fixed in 4% paraformaldehyde and permeabilized with 0.3% Triton X-100 in PBS. Cells were then blocked for 1 h at room temperature in a solution containing 2% BSA and 5% normal goat serum in PBS-Triton. Coverslips were then incubated overnight at 4 °C in a humidified chamber with primary antibodies.

iPSCs were characterized for pluripotency status via immunocytochemical analysis using the following primary antibodies: anti-Oct4 (Abcam #ab19857, 1:100), anti-SOX2 (Abcam #ab79351, 1:100), anti-Nanog (Abcam #ab80892, 1:100), anti-SSEA-4 (Millipore #MAB4304, 1:100). iMG were characterized using primary antibodies at the following dilutions: anti-IBA1 (Abcam, #ab178846, 1:500), anti-P2RY12 (Sigma Aldrich, #A014518, 1:200), anti-NLRP3 (Santa Cruz, #sc-518123). After washing with PBS, cells were then incubated with Alexa-conjugated anti-rabbit and Alexa-conjugated anti-mouse secondary antibodies (1:500) for 1 h at room temperature. Following another wash step, DAPI (Merck, #MBD0015) was applied for 10 min at room temperature, coverslips were mounted onto glass microscope slides using ProLong™ glass antifade mounting media (Thermo Fisher), and allowed to dry overnight. Images for iMG were captured keeping the settings constant for each antibody using the confocal microscope (Leica TCS SP5, Leica Microsystems, Wetzlar, Germany). Pictures for iPSCs characterization were taken by fluorescence microscope (ECHO Revolve, Discover Echo, San Diego, CA, USA). At least 3 fields of view were analyzed per coverslip.

### Protein extraction and western blot analysis

Cultured iMG were harvested by collecting both floating and adherent iMG into the same tube. The adherent cells were dissociated by incubation with TrypLE for 5 min at 37 °C, then all cells were centrifuged at 250 x g for 5 min. The resulting pellet was washed 1X with PBS then centrifuged again at 500 x g for 10 min for collection. Cell pellets were sequentially homogenized using RIPA lysis buffer (Thermo Scientific) containing protease-phosphatase inhibitor cocktail (Roche). 10ug of each protein sample was run via SDS-PAGE on gradient gels under reducing conditions. Gels were blotted onto polyvinylidene difluoride (PVDF) membranes and blocked with Intercept blocking reagent (Li-Cor) for 1 h at room temperature. Primary antibodies used for western blotting were: anti-GAPDH (Millipore #MAB374, 1:10,000) anti-IBA1 (Abcam #ab178846, 1:500), anti-PLIN3 (Proteintech #10694-1-AP, 1:500), and anti-iNOS (Proteintech #18985-1-AP, 1:500). Blots were washed 3x with 1X TBS-T, probed with the appropriate fluorescent secondary antibody (IRDye, Li-Cor), washed 3x with 1X TBS-T and imaged with the Odyssey XF Dual-Mode Imaging System (Li-Cor).

### LPS and neuromelanin treatment of iMG

iMG were plated at a density of 50,000 cells/cm2 on uncoated 6-well plates and cover slips. After allowing the cells to attach for 24 h, they were treated with 100 ng/ml of LPS (Thermo Scientific) for 24 h. For western blot analysis, both floating and adherent iMG were collected into the same tube. The adherent cells were dissociated by incubation with TrypLE for 5 min at 37 °C, then all cells were centrifuged at 250 x g for 5 min. The resulting pellet was washed 1X with PBS then centrifuged again at 500 x g for 10 min for collection.

For treatment with neuromelanin, mature iMG were plated at a density of 50,000 cells/cm² on uncoated 24-well plate coverslips and allowed to attach for 24 h. Neuromelanin suspension was prepared from a purified human neuromelanin stock (0.5 mg/ml) stored at − 80 °C. Neuromelanin was sterilized using a standard ethanol-based procedure, dried, and subsequently resuspended in sterile water to the desired working concentration. To ensure a uniform suspension, the material was gently dispersed using mild mechanical agitation. The neuromelanin was allowed to fully rehydrate over several days at room temperature and prior to treatment, aliquots were mixed carefully. The neuromelanin suspension was pre-incubated in complete culture medium for 2–3 h to allow interaction with medium components before being applied to the cells at a final concentration of 5 µg/mL for 24 h. Brightfield images were acquired using a Nikon Eclipse Ts2 inverted microscope equipped with a TrueChrome Metrics digital camera.

### Targeted transcriptomic analysis using the NanoString neuroinflammation panel

Targeted gene expression profiling was performed using the nCounter Human Neuroinflammation Panel v1.0 (NanoString Technologies, Seattle, WA, USA), which includes 770 curated mRNA probes related to key neuroinflammatory pathways.

Total RNA was extracted from frozen iMG pellets using the RNeasy Mini Kit (Qiagen, Hilden, Germany) following the manufacturer’s protocol, and samples were diluted to a final concentration of 20 ng/µL. For each reaction, 100 ng of RNA was hybridized with capture and reporter probes overnight at 65 °C for 16 h.

Following hybridization, samples were transferred to German Cancer Consortium (DKTK) core facility, where post-hybridization processing and data acquisition were performed using the nCounter Analysis System (NanoString Technologies), in accordance with the manufacturer’s instructions. Raw digital count data were analyzed using nSolver™ Analysis Software (v4.0) and the Advanced Analysis module.

Normalization of mRNA counts was carried out using NanoString’s automated reference gene selection method, which identifies a subset of the most stable housekeeping genes from the panel. Background correction was based on the included negative control probes, and all samples met quality control criteria. The resulting normalized counts were exported for downstream statistical analysis. For integration of the two BPAN and control cell lines, the data were filtered to retain genes with at least three quantification values per cell line. Subsequently, an ordinary least-squares linear model was fitted to estimate the genotype effect while controlling for cell line for each gene using Python (v3.12) with the package pandas (v2.2.3), numpy (v1.26.4), and statsmodels (v0.14.4). P-values were adjusted for multiple testing using the Benjamini–Hochberg false discovery rate (FDR) method.

Given the limited number of donor lines (two BPAN patient and two control lines), genotype is inherently confounded with cell line. Therefore, model estimates are interpreted as comparisons between BPAN patient and control groups at the level of donor lines, rather than independent population-level genotype effects.

Pathway-level analysis was performed using the panel’s integrated pathway scoring algorithm, which calculates the first principal component of gene sets associated with each predefined neuroinflammatory pathway to provide an overall pathway activity score per sample.

### Secretomics

Cells were culture in 50 µM N-azido‐mannosamine (ManNAz) for 48 h. The media was collected and filtered with a 0.22 μm spin filter (Costar Spin-X, Sigma Aldrich, CLS8160).

The supernatants were washed two times with 250 µL PBS on 10 K Vivacon filters (Sartorius, Germany) and concentrated to 50 µL. Biotinylation of labeled glycoproteins was performed by adding 5 µL 10 mM Sulfo-dibenzocyclooctyne-biotin (Sigma Aldrich, 760706) and incubation for 16 h at 4 °C while shaking at 750 rpm. Afterwards, samples were washed three times with PBS, concentrated to 50 µL and recovered by reverse spinning.

The supernatants were subjected to a lectin-based enrichment using 50 µL concanavalin A (ConA) beads (Merck, C7555) per sample. Glycoproteins were bound to the beads in 1 mL binding buffer (5 mM MgCl_2_, 5 mM MnCl_2_, 5 mM CaCl_2_, 0.5 M NaCl, 20 mM Tris-HCl pH 7.5) rotating end-over-end for at least 2 h at 4 °C followed by two washing steps with 1 mL binding buffer. Proteins were eluted in 500 µL elution buffer (500 mM Methyl-α-D-mannopyranoside, 10 mM EDTA, 20 mM Tris-HCl pH 8.5) and Triton X-100 was added to a final concentration of 1% (v/v).

Glycoprotein enrichment was performed with 10 µL of magnetic Streptavidin beads (Thermo Fisher Scientific, 88817) per sample. Beads were washed three times with 1 mL 1% (v/v) Triton-X before they were added to the samples. Then, samples were incubated rotating end-over-end for 2 h at 4 °C. The streptavidin beads with the bound glycoproteins were washed three times with SDS buffer (1% (w/v) SDS, 250 mM NaCl, 100 mM Tris-HCl pH 8.5), three times with urea buffer (3 M urea, 0.1% sodium deoxycholate (SDC), 50 mM ammonium bicarbonate (ABC) and once with 0.1% SDC in 50 mM ABC using a Dynamag 2 magnetic rack (Thermo Fisher Scientific, US).

Protein reduction was performed with 100 µL 10 mM dithiothreitol in 0.1% SDC and 50 mM ABC and incubation for 30 min at 37 °C while shaking at 1000 rpm. The supernatant was discarded and protein alkylation was performed with 100 µL 40 mM iodoacetamide in 0.1% SDC and 50 mM ABC and incubation for 30 min at 24 °C in the dark while shaking at 1000 rpm. After removal of the supernatant, on-bead protein digestion was performed in 60 µL 0.1% SDC and 50 mM ABC with 0.2 µg LysC (Promega) for 3 h at 37 °C and subsequently with 0.2 µg trypsin (Promega) for 16 h at room temperature.

The supernatant was collected and peptides were purified adding 200 µg of a one-to-one mixture hydrophilic and hydrophobic carboxylate coated SeraMag speedbeads (Cytiva, US) using a magnetic rack. Peptide retention on the beads was facilitated by adding acetonitrile (Sigma Aldrich) to a final concentration of more than 95% (v/v) followed by one washing step with 100% (v/v) acetonitrile. After removal of the supernatant, Peptides were eluted in 40 µL 0.1% (v/v) formic acid (FA, Sigma Aldrich) and filtered through 0.22 μm spin filters (Costar Spin-X, Sigma Aldrich, CLS8160). Samples were dried by vacuum centrifugation and dissolved in 20 µL 0.1% formic acid directly before starting the mass spectrometric analysis.

### Mass spectrometry

A volume of 8 µL per sample was separated on a nanoElute nanoHPLC system (Bruker, Germany) on an in-house packed C18 analytical column (15 cm × 75 μm ID, ReproSil-Pur 120 C18-AQ, 1.9 μm, Dr. Maisch GmbH) using a binary gradient of water and acetonitrile (B) containing 0.1% formic acid at flow rate of 300 nL/min (0 min, 2% B; 2 min, 5% B; 62 min, 24% B; 72 min, 35% B; 75 min, 60% B) and a column temperature of 50 °C. The nanoHPLC was online coupled to a TimsTOF pro mass spectrometer (Bruker, Germany) with a CaptiveSpray ion source (Bruker, Germany). A Data Independent Acquisition Parallel Accumulation–Serial Fragmentation (diaPASEF) method was used for spectrum acquisition. Ion accumulation and separation using Trapped Ion Mobility Spectrometry (TIMS) was set to a ramp time of 100 ms. One scan cycle included one TIMS full MS scan with 26 windows with a width of 27 m/z covering a m/z range of 350–1001 m/z. Two windows were recorded per PASEF scan. This resulted in a cycle time of 1.4 s.

### Mass spectrometry data analysis

The software DIA-NN version 1.8.1 was used to analyze the data^80^. The raw data was searched against a one protein per gene database from homo sapiens (UniProt, 21758 entries, download: 2024-07-30) and a database with potential contaminants (246 entries) from Maxquant^81^ using a library free search. Trypsin was defined as protease and two missed cleavages were allowed. Oxidation of methionines and acetylation of protein N-termini were defined as variable modifications, whereas carbamidomethylation of cysteines was defined as fixed modification. Variable modifications were restricted to two per peptide. The precursor and fragment ion m/z ranges were limited from 350 to 1001 and 200 to 1700, respectively. Precursor charge states were set to 2–4 charges. An FDR threshold of 1% was applied for peptide and protein identifications. The mass accuracy for MS1 and MS2 were set to 15 ppm. Ion mobility windows were automatically adjusted by the software. The match between runs and RT-dependent cross-run normalization options were enabled.

The software Perseus version 1.6.2.3^82^ was used for further data analysis. Contaminants were removed. Protein label-free quantification (LFQ) intensities were log2 transformed. One LC–MS/MS run (8172-14_run3) was excluded due to technical problems according to quality-control assessment showing a very low peptide identification rate and strong deviations in LFQ intensity distribution relative to other runs. Changes in protein abundances between the different groups were calculated and a two-sided Student’s t-test was applied comparing the log2 transformed LFQ intensities between the groups. At least three valid values per group were required for statistical testing.

For integration of the two BPAN patient and control cell lines, the data was filtered for proteins with at least three quantification values per cell line. Afterwards, an ordinary least-squares linear model was fitted to estimate the genotype effect while controlling for line for each protein using Python (v3.12) with the packages pandas (2.2.3), numpy (1.26.4) and statsmodels (v0.14.4). A Benjamini-Hochberg FDR correction was applied. Given the limited number of donor lines (two BPAN patient and two control lines), genotype is inherently confounded with line; therefore, model estimates are interpreted as line-level contrasts grouped by genotype rather than independent population-level genotype effects.

### Pathway enrichment analysis for secretomics

Differentially expressed proteins were defined by a p-value < 0.05 for both BPAN patient lines versus both controls. Pathway enrichment analysis was performed using the web-based program DAVID version 6.8 and the Functional Annotation Clustering tool. Gene lists for either increased- or reduced proteins were compared to all proteins relatively quantified in LC-MS/MS in all cell lines, defined as experimental background.

### Cross-layer integration analysis

RNA differential-expression results from the NanoString panel were integrated with secretome proteomics data by restricting analysis to genes quantified in both datasets. Multiple-testing correction for both RNA and protein analyses was performed using the Benjamini–Hochberg (BH) FDR method. Integrated findings were classified into tiers:

Tier 1 (Confirmatory): Genes meeting BH-adjusted q < 0.10 independently in both RNA and protein layers.

Tier 2 (Exploratory integrated): Genes meeting BH-adjusted q < 0.05 for a combined RNA–protein statistic derived using a directional Stouffer method (see below), and exhibiting concordant direction of log2 fold change across layers.

Tier 3: Genes quantified in both datasets but not meeting Tier 1 or Tier 2 criteria.

### Directional stouffer integration

Two-sided p-values from RNA and protein analyses were converted to z-scores using the inverse normal transformation. Each z-score was multiplied by the sign of the corresponding log2 fold change to encode directionality. Directional z-scores from RNA and protein layers were combined using equal-weight Stouffer integration. Combined two-sided p-values were calculated from the resulting Z statistic and adjusted using BH-FDR across the shared gene universe to obtain Combined_q values. To assess whether nominal overlap (*p* < 0.05 in both layers) exceeded chance expectation, Fisher’s exact (hypergeometric) test was performed within the shared gene universe. To evaluate whether RNA–protein concordance exceeded random pairing, protein log2 fold-change values were randomly permuted across genes (10,000 iterations), and Spearman correlation coefficients were recomputed to generate a null distribution.

### Statistical analysis

Student’s *t*-test analysis and ANOVA followed by Tukey’s post-hoc test was performed. *P*-values less than 0.05 were considered significant. All errors bars shown in the figures are standard error of the mean (SEM) and asterisks denotes *p* < 0.05 (*), *p* < 0.01 (**) and *p* < 0.001 (***).

### Resource availability

This study did not generate new unique reagents.

## Supplementary Information

Below is the link to the electronic supplementary material.

Supplementary Material 1

Supplementary Material 2

Supplementary Material 3

Supplementary Material 4

Supplementary Material 5

Supplementary Material 6

Supplementary Material 7

## Data Availability

The datasets generated and/or analyzed during the current study are available in public repositories. NanoString gene expression data have been deposited in the Gene Expression Omnibus (GEO) under accession number GSE313884. Secretomic data have been deposited in the PRIDE repository under accession number PXD071930.
