# Supplementary material for: Aberrant immunomodulatory signature in β-propeller protein-associated neurodegeneration patient iPSC-derived microglia
Source: Sci Rep. 2026 Jun 15;16:18516. doi: 10.1038/s41598-026-55648-w (PMC13270018; doi:10.1038/s41598-026-55648-w)
Supplement: Supplementary file 1 — Supplementary Material 1 [file 41598_2026_55648_MOESM1_ESM.pdf]

**SUPPLEMENTAL INFORMATION for**

**Title:** Aberrant immunomodulatory signature in β-propeller protein-associated neurodegeneration patient iPSC-derived microglia

**Authors**: Gamze Özata[1,2*], Rachel M. Wise[1,3*], Aida Cardona-Alberich[1], Naiyareen F. Mayeen[1], Stephan A. Müller[4,5,6], Stefan F. Lichtenthaler[4,5,6], Luigi Zecca[7], Dimitri Krainc[3], Lena F. Burbulla[1,5,6#]

**Correspondence to:** Lena.Burbulla@med.uni-muenchen.de

**This file includes:**

Supplemental Figures S1-S4

Supplementary File – uncropped immunoblots

Legends for Supplementary Dataset Files 1 to 6

**Supplementary Figure 1**

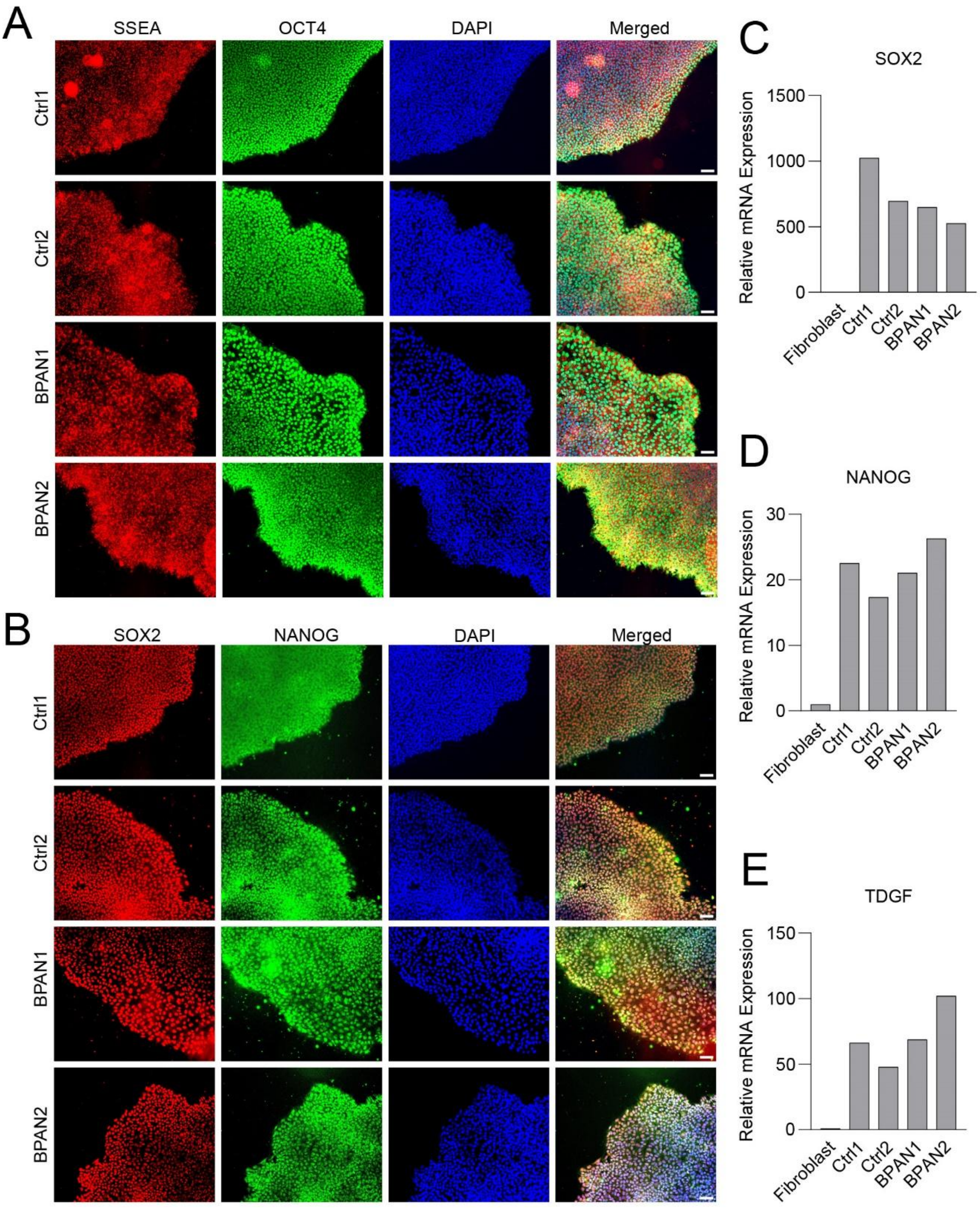


**Supplementary Figure 1. Characterization of iPSC lines from control individuals and BPAN patients.** (**A**) Representative immunofluorescence images of pluripotency markers SSEA4 (red), OCT4 (green), and nuclear counterstain DAPI (blue) in iPSC colonies derived from two control (Control1, Control2) and two BPAN patients (BPAN1, BPAN2). Merged images are shown for each line. Scale bar, 50 µm. (**B**)

Representative immunofluorescence images of SOX2 (red), NANOG (green), and DAPI (blue) staining in the same iPSC lines as in (A), with merged channels. Scale bar, 50 μm. (**C–E**) Quantitative PCR analysis of pluripotency marker expression in iPSC lines relative to parental fibroblasts. (C) Relative mRNA expression of *SOX2* in duplicate samples, normalized to fibroblast levels. (D) Relative *NANOG* expression. (E) Relative *TDGF* expression.

**Supplementary Figure 2**

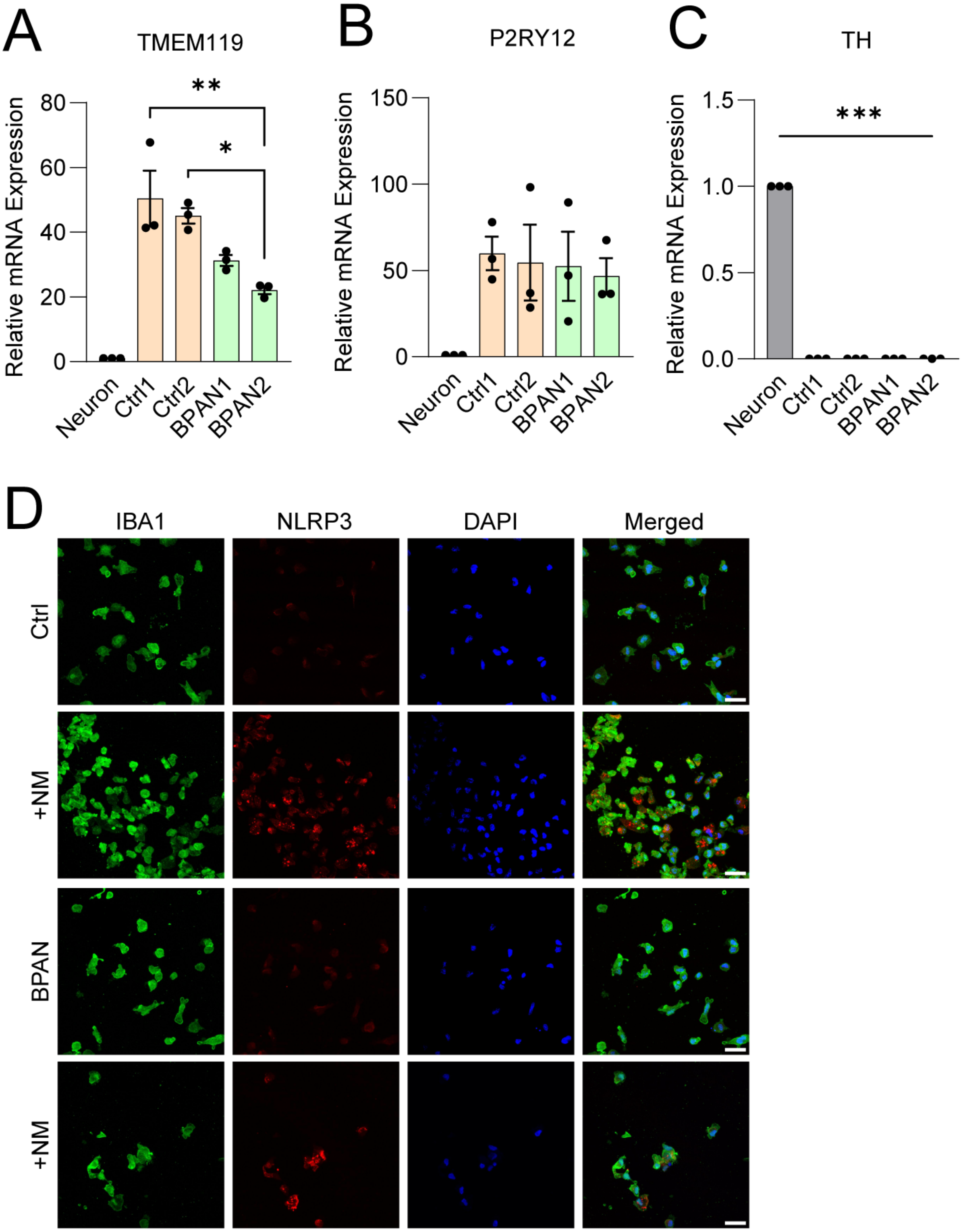


**Supplementary Figure 2. Characterization of iPSC-derived microglia culture.** (**A-C**) Quantitative PCR analysis of marker gene expression in iMG compared to midbrain dopaminergic neurons. (A) *TMEM119*, (B) *P2RY12,* and (C) *TH* (tyrosine hydroxylase)

mRNA levels are shown as relative expression (n = 3 independent differentiations). (D) Representative immunofluorescence images of control and BPAN patient iMG stained for IBA1 (green), NLRP3 (red) and DAPI (blue), with and without neuromelanin treatment (5 µg/mL, 24 hours). Scale bar, 10 µm. NM, neuromelanin.

## Supplementary Figure 3

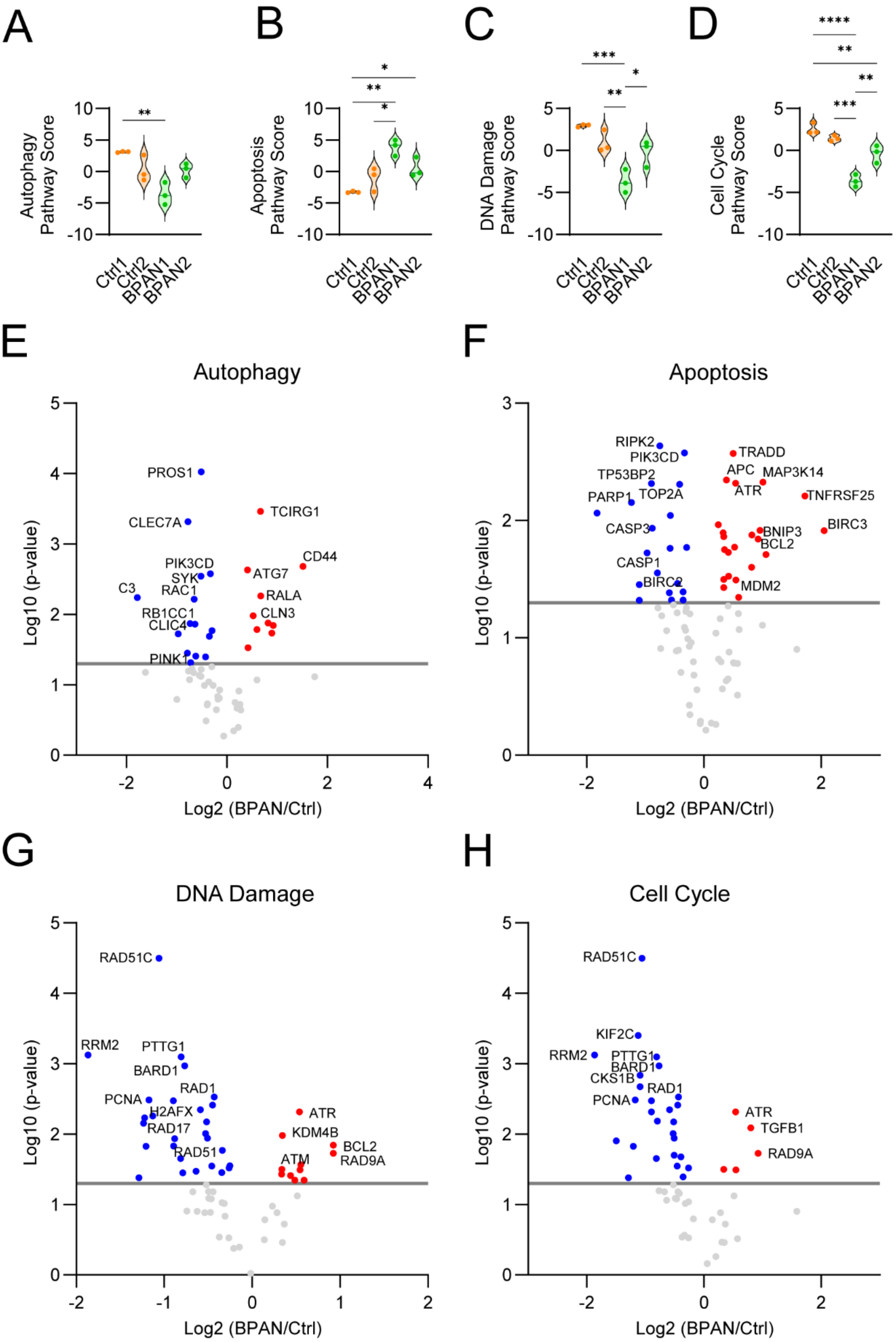


**Supplementary Figure 3. NanoString-based neuroinflammatory pathway analysis of iPSC-derived microglia from controls and BPAN patients.** (**A-D**) Violin

plots showing pathway scores for selected functional categories, including autophagy (A), apoptosis (B), DNA damage (C), cell cycle (D). Each violin plot displays the distribution of pathway scores for all samples in each group (n = 3 independent differentiations with 2 iPSC lines per control/patient group). Statistical significance was determined by one-way ANOVA. (**E-H**) Volcano plots illustrating differential gene expression within the most relevant pathways: autophagy (E), apoptosis (F), DNA damage (G), cell cycle (H). Red dots indicate upregulated genes, blue dots indicate downregulated genes, and light gray dots represent nonsignificant changes. The gray horizontal line marks the significance threshold ($p$ = 0.05, $-\log_{10}$ = 1.3).

**Supplementary Figure 4**

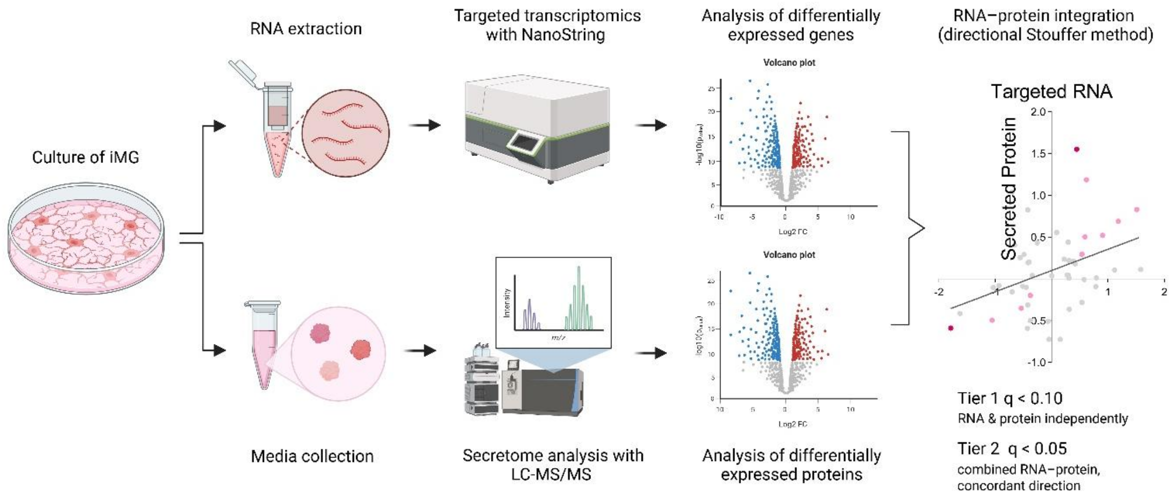


**Supplementary Figure 4. Integrated transcriptomic and secretomic workflow with tiered RNA-protein analysis.** Schematic overview of the experimental and analytical pipeline for integrated transcriptomic and secretomic analyses. Induced microglia (iMG) derived from BPAN patient and control iPSC lines were cultured, followed by RNA extraction and conditioned media collection. Targeted transcriptomic profiling was performed using the NanoString Neuroinflammation panel, and secreted proteins were analyzed by LC-MS/MS-based secretomics. Differential expression analyses were conducted independently for RNA and protein datasets (volcano plots). Cross-layer integration was performed on genes quantified in both datasets using a directional Stouffer method. Genes were classified into tiers: Tier 1 (confirmatory), defined by Benjamini-Hochberg (BH) adjusted $q < 0.10$ independently in both RNA and protein layers; and Tier 2 (integrated), defined by BH adjusted $q < 0.05$ for the combined RNA-protein statistic and concordant $\log_2$ fold-change direction across layers. Arrows indicate direction of regulation. Created in BioRender. Oezata, G. (2026) https://BioRender.com/7oet6xf.

**Supplementary File – uncropped immunoblots**

Immunoblot – Figure1B

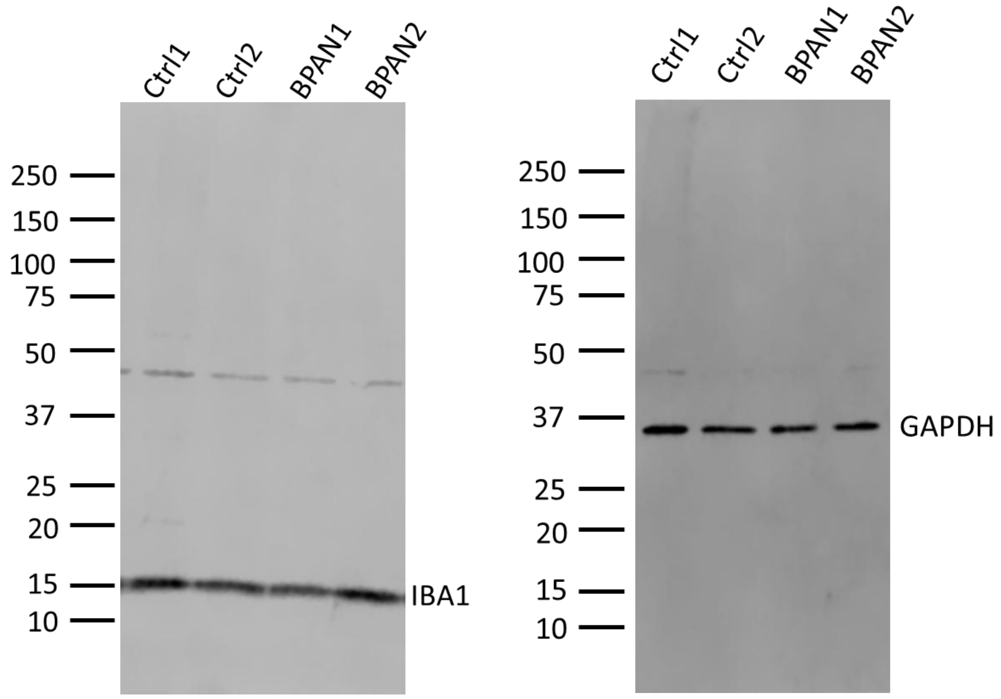


Immunoblot - Figure 1C

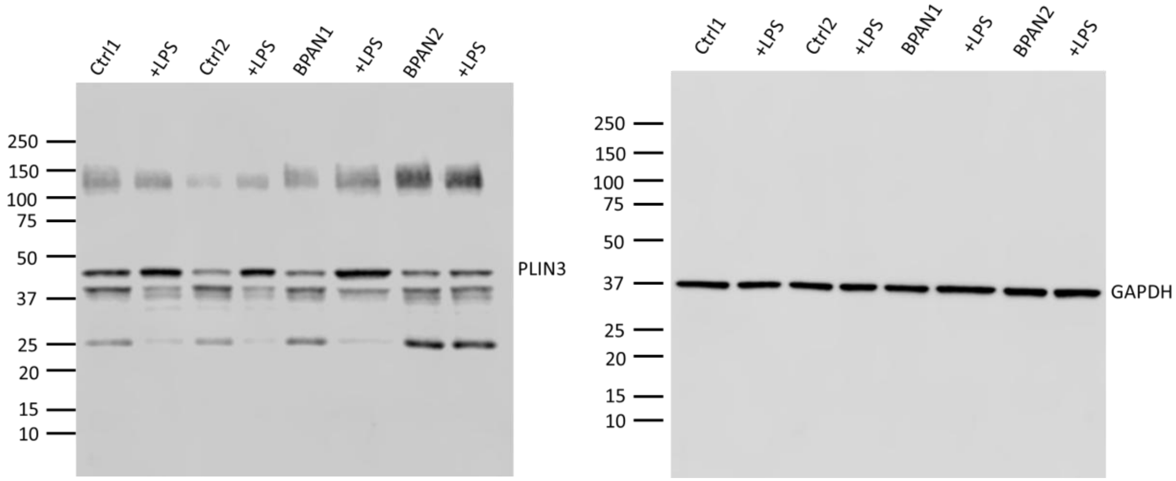

Immunoblot – Figure1D

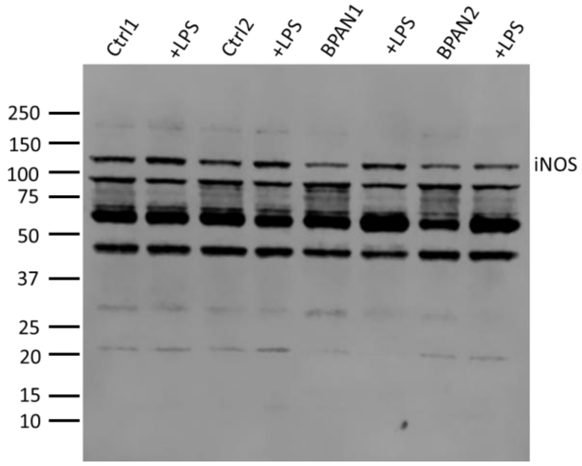


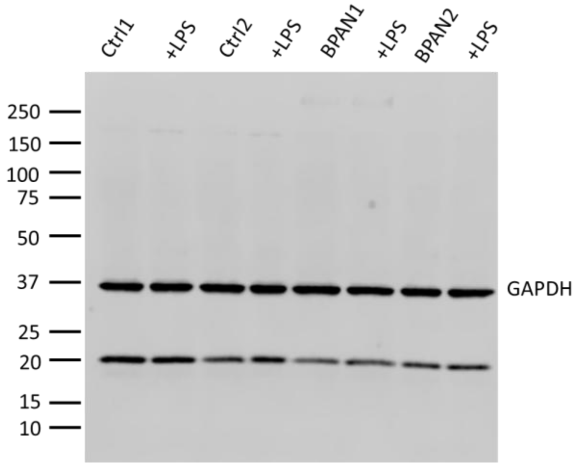

**Supplementary Dataset File 1**. List of donor-derived cell lines

* see attached Excel file "Supplementary Dataset File 1"

**Supplementary Dataset File 2**. Targeted transcriptomics data from Nanostring platform on iMG from BPAN patients and healthy control lines

* see attached Excel file "Supplementary Dataset File 2"

**Supplementary Dataset File 3**. Comparison of targeted transcriptomics on iMG from BPAN patients and healthy control lines to a published sex-differential microglial gene signatures data set (Guneykaya, D. *et al.,* 2018*)*

* see attached Excel file "Supplementary Dataset File 3"

**Supplementary Dataset File 4**. Secretomics data using high-performance secretome protein enrichment with click sugars (hiSPECS) on iMG from BPAN patients and healthy control lines and list of stratified secretome proteins based on curated UniProt annotations

* see attached Excel file "Supplementary Dataset File 4"

**Supplementary Dataset File 5**. Integration of targeted transcriptomic and secretomic data with defined log2 fold-change values in both datasets

* see attached Excel file "Supplementary Dataset File 5"

**Supplementary Dataset File 6**. List of clusters resulting from pathway enrichment analysis of secreted proteins from BPAN patient and control iMG

* see attached Excel file "Supplementary Dataset File 6"
